# 

**Published:** 1908-10

**Authors:** 


### CONTENTS.

ORIGINAL COMMUNICATIONS—

Quacks, by M. A. Clark, M. D., Macon, Ga .......... 353
Exploratory Puncture of the Drum Membrane In Certain Diseases of the Middle Ear. By Dunbar Roy, M. D., Atlanta, Ga. .......... 158
The Treatment of the Morphine Habit in General Practice.—Morphinomani—The Morbid Desire for Morphine—The Opium Habit. By Geo. H. Lehmann, Augusta, Ga. .......... 365
Conservatism In Railway Surgery. By W. P. Whittington, M. D., Asheville, N. C. .......... 371
Empyema: Etiology, Symptoms, Treatment and When to Perform Thoracotomy, Report of Cases. By John T. Burrus, M. D., High Point, N. C. .......... 374
Report of a Case of Tropical Ulcer. By Dr. W. E. Welmerding, Atlanta, Ga. .......... 379

EDITORIALS .......... 381
NEWS AND NOTES .......... 389
BOOK REVIEWS .......... 399

